# Association Between Dietary Animal Protein and Depression in a General Population

**DOI:** 10.3390/nu18071104

**Published:** 2026-03-30

**Authors:** Sunghee Lee

**Affiliations:** Department of Food and Nutrition, College of Health Science, Kangwon National University, Samcheok 25945, Gangwon, Republic of Korea; sunglee@kangwon.ac.kr

**Keywords:** animal protein, depression, general population, plant protein, protein intake, psychological disorder

## Abstract

Background/Objectives: Because of conflicting evidence about the effects of protein sources on mental health, this study aimed to investigate whether animal or plant protein intake is associated with depression. Methods: Among 17,125 adults (7287 men and 9838 women) from nationally representative survey data, the primary measure of depression was determined with the Patient Health Questionnaire-9. A 24-h recall method was employed for dietary assessment. The associations between tertile ranges of animal or plant protein and depression were analyzed with weighted logistic models, adjusted for potential confounders. Results: Despite no association among men, women in the uppermost tertile of animal protein intake demonstrated a 36% lower likelihood of having depression [95% confidence interval 0.48–0.86]. Additionally, participants older than 65 years presented a significant trend toward lower likelihoods of depression associated with animal protein intake. Conclusions: This large cross-sectional study of the general population revealed that, regarding one of the common psychological disorders—depression—animal protein intake might have a beneficial association.

## 1. Introduction

Depression is a highly common mental health issue and one of the most pervasive psychiatric disorders affecting individuals’ overall well-being. Approximately 5% of the general population has major depression [1]. Women are affected more than men—6% of women compared with 4% of men [1]. This burden is even greater among older adults, with a 5.7% prevalence in individuals aged over 60 years. Depression can affect various aspects of individuals’ lives, resulting in an elevated risk of metabolic syndrome [2] and Alzheimer’s disease [3]. Furthermore, a meta-analysis indicated an increased risk of suicidal events [4].

Establishing dietary guidelines that target modifiable risk factors is essential for the prevention and management of depression. Depression has been linked to the intake of specific dietary amino acids, particularly tryptophan, a serotonin precursor that plays an important role in mood-regulatory pathways [5]. Notably, tryptophan intake exhibited an inverse association with depression [6]. Furthermore, a study involving 17,845 individuals demonstrated a notable inverse association between total protein and depression (OR = 0.34, 95% CI 0.17–0.68) [7]. Correspondingly, lower protein intake revealed a markedly increased risk of depression, as evidenced by findings from both Korea (OR = 3.17, 95% CI 1.60–6.29) and the United States (OR = 1.65, 95% CI 1.18–2.30) [8].

Little research has been conducted on the relationship between animal protein (AP) intake as well as plant protein (PP) intake and depression, with one study showing that adults with higher AP intake had lower odds of depression [9]. Specifically, men in the uppermost quintile of AP exhibited a significantly decreased likelihood of experiencing depression (OR = 0.66, *p* for trend = 0.009) [9]. Another meta-analysis suggested that meat consumption had a significant association with a decreased burden of depression [10]. Similarly, a study of 23,313 individuals indicated an inverse association between AP intake and depression (OR = 0.60, 95% CI 0.45–0.80) [11]. However, contrary evidence has also been reported; a study found that high AP was associated with an increased likelihood of depression among 489 women (OR = 2.63 95% CI 1.45–4.71), whereas PP showed no significant association [12]. Nevertheless, most contradictory findings regarding PP have primarily related to cardiometabolic effects rather than psychological outcomes.

Therefore, this study focused on investigating whether AP or PP is associated with depression in 17,125 adults (7287 men and 9838 women) using the Korea National Health and Nutrition Examination Survey (KNHANES).

## 2. Methods

### 2.1. Study Participants

The KNHANES, a national survey, has been performed to monitor health status and nutritional intake of the Korean general population [13]. This study utilized the publicly available secondary database and was conducted through large cross-sectional analyses. All participants in this survey completed questionnaires regarding health-related risk factors, underwent venipuncture for blood sample collection, and received physical examinations [13]. Alcohol drinking status was classified as either consuming alcohol more than once a month in the previous year or less than once per month [13]. From the Global Physical Activity Questionnaire (GPAQ), physical activity (PA) was evaluated based on the intensity and duration of activities at work, recreational activities, travel, and walking [14,15]. The Korean version was validated and confirmed to be reproducible [15]. The ‘active’ status of PA was identified with ≥150 min a week as moderate activities, ≥75–150 min a week as vigorous activities, or an analogous combination [16]. Otherwise, PA was classified as ‘inactive’ [16]. Income was categorized by quartile ranges (Q1–Q4) [13]. Hypertension was determined by anti-hypertensive medication, ≥140 mmHg systolic blood pressure (BP), or ≥90 mmHg diastolic BP. Additionally, diabetes was identified with anti-diabetic medication or ≥126 mg/dL fasting glucose. All participants agreed and completed an informed consent form. This study complied with the Declaration of Helsinki and received approval from an Institutional Review Board (KWNUIRB-2025-08-020).

**Figure 1:**
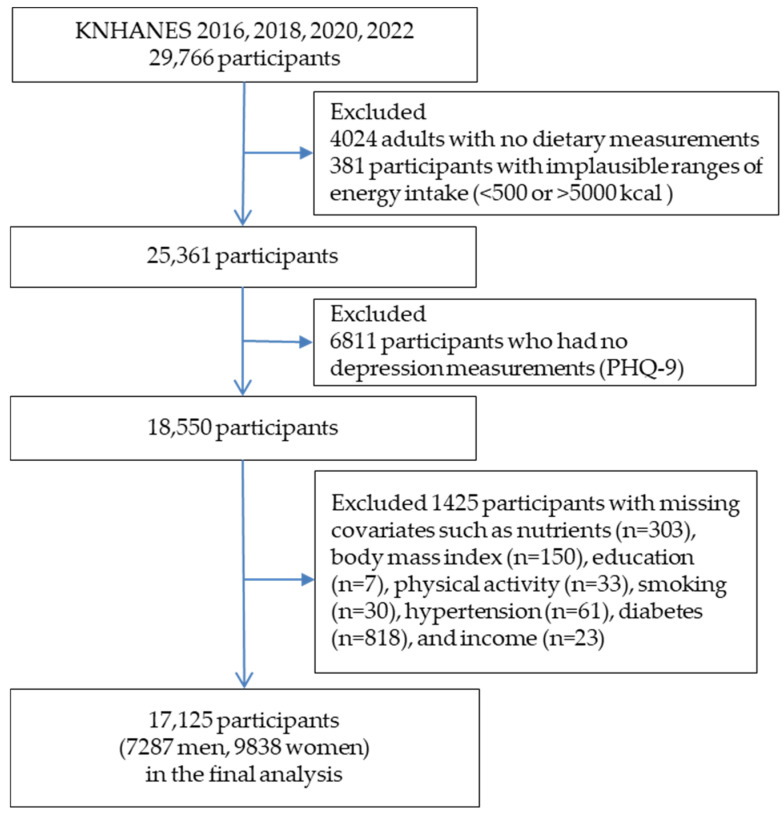
Flow diagram of the study participants.

Since the primary outcome measurement, depression, was assessed every other year, 29,766 individuals from the 2016, 2018, 2020, and 2022 KNHANES surveys were included. Of these, 4024 and 381 participants were excluded due to a lack of dietary examination and implausible energy intake outside the range of <500 or >5000 kcal, respectively. Furthermore, 6811 participants were excluded because they had no measurements for the Patient Health Questionnaire (PHQ-9). Subsequently, among these 18,550 individuals, 1425 were excluded because of missing covariates for specific nutrients (*n* = 303), smoking status (*n* = 30), PA (*n* = 33), body mass index (BMI) (*n* = 150), education (*n* = 7), hypertension (*n* = 61), income (*n* = 23), and diabetes (*n* = 818). Ultimately, 17,125 participants were included. The flowchart is shown in Figure 1.

### 2.2. Depression Ascertainment

Depression cases were identified based on the PHQ-9—a diagnostic tool that involves a screening test [17,18]. Usually, adults with a score of ≥10 out of 27 were identified as having depression [19]. The reliability as well as validity of the Korean version were verified [20]. As for the internal consistency of the PHQ-9, the Cronbach’s alpha value was 0.79.

### 2.3. Dietary Assessment

With guidance from a professionally trained interviewer, all individuals completed a 24-h recall test. Each food item and its dietary nutrients were estimated using the up-to-date database of the Rural Development Administration of Korea [21]. To investigate the proportion of deficient protein, two cutoff were used: <0.8 g/kg/day protein intake according to the World Health Organization [22] and the recommended dietary intake (RDI) based on age groups and sex according to the Korean Dietary Guidelines [23]. The current RDIs are 65 g/d for men aged 19–49 years, 60 g/d for men aged >50 years, 55 g/d for women aged 19–29 years, and 50 g/d for women aged >30 years [23].

### 2.4. Statistical Analyses

Considering the complex sampling for the KNHANES data, weighted survey methods were employed for all the analyses. To differentiate general characteristics related to protein intake between participants with and without depression, weighted survey means and frequencies were used with survey frequency and regression models. Additionally, the general characteristics of health-related risk factors were compared according to the tertile ranges of protein intakes. To investigate the associations, survey logistic regression was conducted, after adjusting for age (continuous, ages), energy (continuous, kcal/day), sex (categorical, men/women), PA (active/inactive), BMI (continuous, kg/m^2^), drinking (continuous, %), marital status (married/single), smoking (categorical, current/ex-/non-smoker), income (categorical, Q1/Q2/Q3/Q4), education (categorical, <high school/high school/>high school), diabetes (categorical, yes/no), and hypertension (categorical, yes/no). Analyses were completed using SAS 9.4 (SAS Institute, Cary, NC, USA) at a *p*-value cutoff of <0.05.

## 3. Results

Table 1 presents a comparison of general characteristics among 17,125 participants with and without depression. Among all participants, those with depression were younger (*p* = 0.012) and had a higher BMI (*p* = 0.030). Depression was also associated with marital status (*p* < 0.001), income (*p* < 0.001), education (*p* < 0.001), smoking (*p* < 0.001), and diabetes (*p* < 0.001). Furthermore, sex-specific results were examined. Interestingly, all participants with depression indicated a significantly lower intake of total protein (*p* < 0.001), animal protein (*p* < 0.001), and plant protein (*p* < 0.001). Moreover, those with depression demonstrated higher percentages of protein deficiency, defined as both < 0.8 g/kg/day (*p* < 0.001) and below RDI (*p* < 0.001). This was observed in men. However, women did not show any significant difference in total protein, animal protein, or PP.

Table 2 exhibits the general characteristics of 7287 men. Men in the highest tertile of AP were relatively younger (*p* < 0.001) and had a higher BMI (*p* < 0.001). They also showed associations with education (*p* < 0.001), PA (*p* < 0.001), alcohol consumption (*p* < 0.001), smoking status (*p* = 0.003), hypertension (*p* < 0.001), marital status (*p* < 0.001), diabetes (*p* < 0.001), and income (*p* < 0.001). Conversely, men in the highest tertile of PP intake were relatively older (*p* < 0.001) and showed significant associations with PA (*p* = 0.005), smoking (*p* = 0.005), and marital status (*p* < 0.001).

Table 3 presents the characteristics of 9838 women. Women in the highest tertile of AP were significantly younger (*p* < 0.001), whereas women in the highest tertile of PP were significantly older (*p* < 0.001). Furthermore, animal protein intake among women was associated with education (*p* < 0.001), PA (*p* < 0.001), smoking (*p* = 0.012), drinking (*p* < 0.001), marital status (*p* < 0.001), income (*p* < 0.001), diabetes (*p* = 0.023), and hypertension (*p* = 0.001). In contrast, plant protein intake among women was associated with education (*p* = 0.006), smoking (*p* < 0.001), drinking (*p* < 0.001), income (*p* = 0.004), marital status (*p* < 0.001), diabetes (*p* < 0.001) and hypertension (*p* < 0.001).

Table 4 highlights the associations of AP and PP with depression among 17,125 participants. Particularly, those in the highest tertile of AP were 30% less likely to have depression (95% CI 0.54–0.90, *p* for trend = 0.006) even after adjusting for potential confounders. Moreover, women in the highest tertile of AP indicated a 36% reduced likelihood of having depression (95% CI 0.48–0.86, *p* for trend = 0.002). Specifically, total participants in the highest tertile of meat protein exhibited a 24% lower likelihood of having depression (95% CI 0.58–0.99, *p* for trend = 0.043).

Table 5 illustrates which ratio of AP to PP intake provides the lowest OR for depression. Participants aged over 65 years who had AP intake of 40–60% and PP intake of 40–60% presented a significantly reduced likelihood of having depression (OR = 0.52, 95% CI 0.34–0.81, *p* for trend = 0.010).

## 4. Discussion

The findings reveal that AP intake might be beneficial to mitigate the burden of depression. This may be attributable to the high quality of AP and its rich content of essential amino acids, which may help reduce the likelihood of depression, especially in women with lower overall AP intake. In the present study, total participants in the highest tertile of AP intake were 30% less likely to have depression compared to those in the lowest tertile, after the adjustment of covariates (*p* for trend = 0.006). Notably, women in the highest tertile of AP intake had a 36% lower likelihood of depression (*p* for trend = 0.002). These findings suggest that higher AP intake may be associated with a lower likelihood of depression. Furthermore, the ideal balance between AP and PP intake to mitigate the burden of depression was investigated. Not surprisingly, regarding the most beneficial ratio, total participants with an AP to PP ratio in the range of 40–60% to 40–60% showed the lowest likelihood of depression in women (OR = 0.63, 95% CI 0.48–0.82). However, even those with a higher proportion of AP at 60–80% with a lower proportion of PP at 20–40% also exhibited a 38% lower likelihood of depression (95% CI 0.54–0.96). Taken together, the present study demonstrates that a modestly higher proportion of AP—rather than a strictly equal balance between AP and PP—may be associated with a lower likelihood of depression, particularly among women and older adults who may be more vulnerable to depressive symptoms.

Little research has investigated the protein sources from AP and PP intake on psychological outcomes such as depression. Consistent with the present study, several studies have reported significant associations between AP intake and a reduced likelihood of common psychological disorders such as depression [9,10,11]. One study in 7169 adults demonstrated that those with higher AP intake had significantly lower likelihoods of depression (OR = 0.73, 95% CI 0.59–0.90; *p*-trend = 0.006) [9]. Particularly, men in the highest quintile of AP intake also had lower odds of depression (OR = 0.66, 95% CI 0.46–0.95; *p*-trend = 0.009) [9]. Additionally, a meta-analysis involving 20 studies indicated that meat consumers showed a significant association with a decreased burden of depression and that vegans had a higher burden of depression than meat consumers [10]. Furthermore, an analysis of 23,313 U.S. adults showed inverse associations of both AP and vegetable PP intake (OR = 0.60, 95% CI 0.45–0.80; OR = 0.61, 95% CI 0.43–0.85, respectively) [11].

Other studies have shown that higher AP intake increases the burden of depression [12,24]. Particularly, the highest tertile of AP intake was linked to a higher likelihood of depressive symptoms among 489 Iranian women (OR = 2.63 95% CI 1.45–4.71) [12]. Additionally, a meta-analysis reported that red and processed meat intake had a significant link to depression (effect size = 1.08, 95% CI 1.04–1.12) [24], suggesting that excessive consumption of red and processed meat may contribute to a higher burden of depression.

The mechanisms underlying the observed link between dietary AP intake and depression need to be elucidated. However, several plausible pathways have been proposed. First, APs are generally rich in tryptophan—an essential amino acid that critically regulates mood as a precursor of serotonin [5,25,26]. As a neurotransmitter, adequate dietary tryptophan can enhance serotonin synthesis, whereas tryptophan deficiency may result in reduced serotonin levels, contributing to depressive symptoms [25,27]. Consistently, previous studies reported that individuals consuming a tryptophan-rich diet have significantly lower odds of depression [27,28]. Generally, AP contains higher levels of tryptophan to favor serotonin synthesis compared to PP [7,29]. Another study of 63,277 participants from the UK Biobank analysis revealed that higher tryptophan intake manifested an inverse association with depression [30]. Notably, the intake of milk/dairy proteins led to a marked reduction in depression among 17,845 adults, suggesting a U-shaped association [7]. Second, AP-rich foods also deliver a range of bioactive micronutrients that are critical for neuropsychological health. Minerals commonly abundant in AP—including heme-iron, vitamin B12, zinc, and calcium—are involved in neurotransmitter synthesis, neuromodulation, and broader brain function [25]. Deficiencies in these micronutrients have been associated with depressive symptoms. For example, inadequate intakes of magnesium, iron, zinc, or calcium have been reported in individuals with depression, particularly in populations prone to nutritional deficiencies [25]. A study of women older than 65 years identified significant links between mineral deficiencies and increased depressive symptoms [31]. Supporting these observations, a meta-analysis showed that higher dietary magnesium was linked to a reduced risk of depression [32]. Additional evidence has also suggested an inverse relationship between magnesium status and depression, underscoring its potential role in mood regulation [33]. Similarly, lower zinc and iron levels have been linked to depressive symptoms in observational studies, highlighting the importance of adequate mineral intake for mental health [25]. Furthermore, animal source foods are major constitutors of certain vitamins—such as vitamin B_12_ and vitamin D—that support neurotransmitter synthesis and immune function; deficiencies in these vitamins have also been associated with depression [25]. Taken together, the potential association between AP intake and depression may reflect not only its favorable essential amino acid profile, such as tryptophan-mediated serotonergic pathways, but also its diverse neurotransmitter source with critical micronutrients essential for psychological health.

This investigation possesses several advantages. First, it utilized a large Asian sample of 17,125 participants, which enabled us to conduct sex-specific examinations and to detect the differences. Second, the use of nationally representative general-population data enhances external validity and supports the generalizability of the findings. Despite its strengths, this study is not without constraints. This cross-sectional study design was inherently limited in its ability to confirm causality. However, overall, it is still worth investigating this association within a cross-sectional study, given that there is very little evidence on depression linked to AP in the general population.

## 5. Conclusions

In conclusion, the findings, based on 17,125 participants from a general population, demonstrated a significantly inverse association with AP intake on the likelihood of depression, especially in women, even after adjusting for potential confounders. Taken together, when it comes to psychological disorders such as depression, AP intake appeared to be beneficial to mitigate the burden of depression. Future prospective studies are required to confirm the associations linking specific amino acids to mood regulation.

## Figures and Tables

**Table 1:** General characteristics according to depression (*n* = 17,125).

		Total (*n* = 17,125)			Men (*n* = 7287)			Women (*n* = 9838)		
		Depression	No Depression	*p*	Depression	No Depression	*p*	Depression	No Depression	*p*
		(*n* = 804, 4.46%)	(*n* = 16,321, 95.54%)		(*n* = 233, 3.25%)	(*n* = 7054, 96.75%)		(*n* = 571, 5.67%)	(*n* = 9267, 94.33%)	
Age, years		45.39 ± 0.77	47.32 ± 0.23	0.012	43.29 ± 1.13	46.66 ± 0.28	0.003	46.60 ± 0.94	48.00 ± 0.25	0.136
Body mass index, kg/m^2^		24.47 ± 0.18	24.07 ± 0.04	0.030	25.17 ± 0.36	24.83 ± 0.05	0.352	24.07 ± 0.21	23.28 ± 0.05	<0.001
Education, %										
>High school		35.35	44.68	<0.001	41.13	47.83	0.154	32.04	41.46	<0.001
High school		37.55	36.32		40.07	37.23		36.12	35.40	
<High school		27.10	19.001		18.80	14.95		31.84	23.14	
Physical activity, %										
	Active	44.28	48.01	0.077	49.23	50.76	0.685	41.45	45.20	0.159
	Inactive	55.72	51.99		50.77	49.24		58.55	54.80	
Smoking status, %										
Current smokers		28.51	18.18	<0.001	47.90	31.33	<0.001	17.43	4.74	<0.001
Ex-smokers		20.42	23.97		32.00	41.24		13.80	6.31	
Non-smokers		51.07	57.84		20.10	27.42		68.76	88.96	
Current drinker, %		53.42	56.98	0.112	70.22	69.02	0.732	43.82	44.67	0.755
Income, %										
	Q1	37.46	22.97	<0.001	43.01	23.45	<0.001	34.29	22.49	<0.001
	Q2	25.55	24.27		21.35	24.24		27.95	24.31	
	Q3	20.81	25.90		19.05	25.77		21.81	26.04	
	Q4	16.18	26.85		16.59	26.55		15.95	27.16	
Marital status, %										
	Married	65.00	75.60	<0.001	54.83	71.09	<0.001	70.81	80.22	<0.001
	Single	35.00	24.40		45.17	28.91		29.19	19.78	
Hypertension, %		30.95	28.14	0.153	39.26	32.15	0.059	26.20	24.04	0.269
Diabetes, %		16.69	10.42	<0.001	22.03	12.21	<0.001	13.63	8.60	<0.001
Total protein, g/day		64.93 ± 1.48	72.77 ± 0.39	<0.001	76.46 ± 2.61	84.32 ± 0.56	0.004	58.34 ± 1.63	60.96 ± 0.38	0.111
Animal protein, g/day		33.47 ± 1.28	38.80 ± 0.34	<0.001	41.07 ± 2.38	46.17 ± 0.50	0.037	29.12 ± 1.40	31.26 ± 0.32	0.132
Plant protein, g/day		31.46 ± 0.65	33.97 ± 0.16	<0.001	35.39 ± 1.26	38.15 ± 0.24	0.030	29.21 ± 0.71	29.70 ± 0.18	0.499
Protein Deficiency, %										
<0.8 g/kg		39.66	30.35	<0.001	34.31	26.47	0.018	42.71	34.32	<0.001
<RDI *		46.01	36.58	<0.001	38.65	31.63	0.042	50.22	41.64	<0.001

Mean ± SE; * less than recommended dietary intake (RDI) for each age and gender group according to the Korean Dietary Guidelines.

**Table 2:** Characteristics according to animal or plant protein intake among men (*n* = 7287).

		Animal Protein				Plant Protein			
		T1	T2	T3	*p*-Value	T1	T2	T3	*p*-Value
		(*n* = 2380, 28.42%)	(*n* = 2439, 33.56%)	(*n* = 2468, 38.02%)		(*n* = 2397, 34.79%)	(*n* = 2437, 33.25%)	(*n* = 2453, 31.96%)	
Min–Max, g/Day		0–24.11	24.11–47.49	47.49–281.23		0–30.08	30.08–43.20	43.20–143.44	
Age, years		52.90 ± 0.47	47.25 ± 0.38	41.20 ± 0.33	<0.001	44.04 ± 0.39	47.92 ± 0.41	47.87 ± 0.41	<0.001
Body mass index, kg/m^2^		24.48 ± 0.09	24.87 ± 0.09	25.10 ± 0.08	<0.001	24.96 ± 0.09	24.83 ± 0.09	24.74 ± 0.08	0.052
Education, %									
>High school		36.66	49.34	54.27	<0.001	46.80	47.70	48.40	0.159
High school		36.06	36.78	38.74		38.77	35.84	37.27	
<High school		27.28	13.88	7.00		14.43	16.46	14.32	
Physical activity, %									
Active		44.97	49.21	56.33	<0.001	49.19	49.16	53.98	0.005
Inactive		55.03	50.79	43.67		50.81	50.84	46.02	
Smoking status, %									
Current smokers		30.76	30.43	33.97	0.003	33.88	30.63	30.98	0.005
Ex-smokers		44.06	41.76	37.89		37.49	42.74	42.84	
Non-smokers		25.18	27.80	28.14		28.63	26.63	26.19	
Current drinker, %		61.54	68.76	74.95	<0.001	70.06	68.42	68.64	0.469
Income, %									
	Q1	29.68	22.03	21.71	<0.001	24.55	23.75	23.92	0.514
	Q2	23.39	24.58	24.32		23.38	24.66	24.43	
	Q3	25.24	26.25	25.16		27.07	24.80	24.68	
	Q4	21.69	27.13	28.82		24.99	26.80	26.97	
Marital status, %									
	Married	76.95	73.94	62.80	<0.001	63.50	74.00	74.67	<0.001
	Single	23.05	26.06	37.20		36.50	26.00	25.33	
Hypertension, %		40.25	32.81	26.12	<0.001	31.28	33.15	32.78	0.423
Diabetes mellitus, %		17.71	12.15	8.99	<0.001	11.84	13.09	12.69	0.441

Mean ± SE.

**Table 3:** Characteristics according to animal or plant protein intake among women (*n* = 9838).

		Animal Protein				Plant Protein			
		T1	T2	T3	*p*-Value	T1	T2	T3	*p*-Value
		(*n* = 3220, 29.96%)	(*n* = 3291, 33.79%)	(*n* = 3327, 36.25%)		(*n* = 3243, 34.49%)	(*n* = 3277, 33.03%)	(*n* = 3318, 32.48%)	
Min–Max, g/day		0–16.70	16.70–33.06	33.06–237.35		0–22.76	22.76–33.28	33.28–156.66	
Age, years		53.97 ± 0.39	47.65 ± 0.34	43.17 ± 0.31	<0.001	45.09 ± 0.36	49.22 ± 0.36	49.60 ± 0.36	<0.001
Body mass index, kg/m^2^		23.80 ± 0.08	23.14 ± 0.08	23.10 ± 0.08	<0.001	23.27 ± 0.09	23.23 ± 0.08	23.44 ± 0.08	0.143
Education, %									
>High school		27.03	42.20	51.23	<0.001	40.48	40.96	41.38	0.006
High school		32.70	36.55	36.67		37.78	33.46	34.96	
<High school		40.27	21.25	12.10		21.75	25.58	23.65	
Physical activity, %									
Active		39.80	45.00	49.25	<0.001	45.90	43.99	45.02	0.416
Inactive		60.20	55.00	50.75		54.10	56.01	54.98	
Smoking status, %									
Current smokers		5.32	5.78	5.27	0.012	6.21	5.68	4.43	<0.001
Ex-smokers		5.49	6.40	8.07		8.48	5.99	5.63	
Non-smokers		89.19	87.82	86.66		85.31	88.33	89.94	
Current drinker, %		36.33	44.45	51.64	<0.001	48.51	42.49	42.66	<0.001
Income, %	Q1	26.61	22.76	20.66	<0.001	24.50	23.07	21.81	0.004
	Q2	25.03	25.73	22.97		25.84	23.28	24.38	
	Q3	24.91	26.10	26.27		26.01	25.69	25.70	
	Q4	23.45	25.41	30.09		23.65	27.96	28.10	
Marital status, %									
Married		86.81	80.19	73.34	<0.001	73.73	81.57	84.10	<0.001
Single		13.19	19.81	26.66		26.27	18.43	15.90	
Hypertension, %		34.08	23.47	16.62	<0.001	21.54	25.58	25.51	<0.001
Diabetes mellitus, %		13.16	7.66	6.49	<0.001	7.22	9.25	10.27	<0.001

Mean ± SE.

**Table 4:** Associations between different protein sources and depression (*n* = 17,125).

		Dietary Intake			***p*** **-Trend**
		T1	T2	T3	
Animal protein					
Total	*n*	5600	5730	5795	
	with/without depression	348/5252 (5.68%)	229/5501 (4.12%)	227/5568 (3.81%)	
	OR (95% CI)	1.00 (Ref)	0.77 (0.62, 0.95)	0.70 (0.54, 0.90)	0.006
Men	with/without depression	103/2277 (4.26%)	64/2375 (2.76%)	66/2402 (2.92%)	
	min-max, g/day	0–24.10	24.11–47.49	47.49–281.23	
	OR (95% CI)	1.00 (Ref)	0.75 (0.50, 1.12)	0.83 (0.52, 1.30)	0.400
Women	with/without depression	245/2975 (7.02%)	165/3126 (5.46%)	161/3166 (4.74%)	
	min-max, g/day	0–16.70	16.70–33.06	33.06–237.35	
	OR (95% CI)	1.00 (Ref)	0.79 (0.61, 1.01)	0.64 (0.48, 0.86)	0.002
Plant protein					
Total	*n*	5640	5714	5771	
	with/without depression	326/5314 (5.13%)	232/5482 (3.99%)	246/5525 (4.23%)	
	OR (95% CI)	1.00 (Ref)	0.88 (0.70, 1.10)	1.03 (0.79, 1.33)	0.934
Men	with/without depression	96/2301 (3.82%)	73/2364 (3.33%)	64/2389 (2.54%)	
	min-max, g/day	0.30–30.07	30.08–43.19	43.21–143.44	
	OR (95% CI)	1.00 (Ref)	1.11 (0.74, 1.64)	0.96 (0.59, 1.55)	0.917
Women	with/without depression	230/3013 (6.44%)	159/3118 (4.65%)	182/3136 (5.88%)	
	min-max, g/day	0.47–22.76	22.76–33.28	33.28–156.66	
	OR (95% CI)	1.00 (Ref)	0.77 (0.59, 0.99)	1.03 (0.76, 1.38)	0.998
Milk and dairy protein					
total		1.00 (Ref)	0.99 (0.72, 1.36)	1.03 (0.74, 1.42)	0.874
men		1.00 (Ref)	0.82 (0.41, 1.64)	1.41 (0.81, 2.45)	0.222
women		1.00 (Ref)	1.04 (0.72, 1.49)	0.82 (0.56, 1.21)	0.322
Meat protein					
total		1.00 (Ref)	0.73 (0.57, 0.93)	0.76 (0.58, 0.99)	0.043
men		1.00 (Ref)	0.56 (0.36, 0.90)	0.69 (0.43, 1.09)	0.115
women		1.00 (Ref)	0.83 (0.62, 1.12)	0.80 (0.58, 1.11)	0.190
Legume protein					
total		1.00 (Ref)	1.04 (0.80, 1.36)	1.09 (0.85, 1.40)	0.508
men		1.00 (Ref)	1.17 (0.70, 1.95)	1.34 (0.83, 2.19)	0.233
women		1.00 (Ref)	0.97 (0.72, 1.29)	0.98 (0.73, 1.31)	0.870

Adjusted for age, body mass index, energy, education, physical activity, marital status, smoking, drinking, income, hypertension, and diabetes mellitus.

**Table 5:** Ratios between animal vs. plant protein intake and depression (*n* = 17,125).

		Ratio of Protein Intake (%)					
		AP 0–20 PP 80–100	AP 20–40 PP 60–80	AP 40–60 PP 40–60	AP 60–80 PP 20–40	AP 80–100 PP 0–20	*p*-Trend
Total (*n* = 17,125)							
*n*		1947	4390	6100	4090	598	
With/without depression		138/1809 (6.60%)	215/4175 (4.85%)	237/5863 (3.69%)	182/3908 (4.31%)	32/566 (5.20%)	
Protein, g/day		46.00 ± 0.64	58.14 ± 0.48	70.10 ± 0.47	89.23 ± 0.69	120.26 ± 2.48	
Animal protein		5.36 ± 0.12	18.33 ± 0.17	35.34 ± 0.25	61.53 ± 0.51	102.21 ± 2.17	
Plant protein		40.64 ± 0.56	39.81 ± 0.33	34.76 ± 0.23	27.70 ± 0.21	18.05 ± 0.40	
OR (95% CI)		1.00 (Ref)	0.79 (0.60, 1.04)	0.63 (0.48, 0.82)	0.72 (0.54, 0.96)	0.77 (0.47, 1.26)	0.084
Men (*n* = 7287)							
*n*		732	1783	2656	1826	290	
With/without depression		36/696 (4.15%)	67/1716 (4.00%)	60/2596 (2.38%)	58/1768 (3.36%)	12/278 (4.22%)	
Protein, g/day		52.19 ± 1.05	65.96 ± 0.77	79.89 ± 0.67	102.85 ± 0.99	135.69 ± 3.34	
Animal protein		6.25 ± 0.20	20.95 ± 0.28	40.33 ± 0.37	71.07 ± 0.75	115.27 ± 2.96	
Plant protein		45.94 ± 0.90	45.02 ± 0.52	39.56 ± 0.33	31.78 ± 0.30	20.42 ± 0.55	
OR (95% CI)		1.00 (Ref)	1.04 (0.60, 1.79)	0.63 (0.37, 1.09)	0.91 (0.53, 1.56)	0.95 (0.43, 2.11)	0.619
Women (*n* = 9838)							
*n*		1215	2607	3444	2264	308	
With/without depression		102/1113 (8.43%)	148/2459 (5.60%)	177/3267 (5.00%)	124/2140 (5.41%)	20/288 (6.51%)	
Protein, g/day		41.38 ± 0.67	51.27 ± 0.50	60.24 ± 0.52	73.46 ± 0.73	99.86 ± 2.94	
Animal protein		4.69 ± 0.13	16.03 ± 0.18	30.31 ± 0.28	50.48 ± 0.53	84.94 ± 2.57	
Plant protein		36.68 ± 0.58	35.24 ± 0.35	29.93 ± 0.26	22.98 ± 0.24	14.92 ± 0.43	
OR (95% CI)		1.00 (Ref)	0.68 (0.50, 0.93)	0.63 (0.47, 0.85)	0.64 (0.46, 0.91)	0.67 (0.36, 1.26)	0.065
Age group, years							
19–44	*n*	263	1127	2261	2142	436	
	With/without depression	22/241 (7.79%)	56/1071 (4.96%)	108/2153 (5.05%)	115/2027 (5.38%)	23/413 (4.83%)	
	OR (95% CI)	1.00 (Ref)	0.64 (0.35, 1.17)	0.71 (0.41, 1.23)	0.78 (0.46, 1.34)	0.69 (0.34, 1.39)	0.941
45–64	*n*	692	1750	2494	1438	135	
	With/without depression	44/648 (5.52%)	72/1678 (4.61%)	82/2412 (2.49%)	48/1390 (2.66%)	8/127 (6.68%)	
	OR (95% CI)	1.00 (Ref)	1.01 (0.63, 1.61)	0.59 (0.38, 0.91)	0.64 (0.39, 1.05)	1.35 (0.54, 3.34)	0.097
65+	*n*	992	1513	1345	510	27	
	With/without depression	72/920 (7.06%)	87/1426 (5.14%)	47/1298(2.88%)	19/491 (3.23%)	1/26 (4.58%)	
	OR (95% CI)	1.00 (Ref)	0.79 (0.53, 1.18)	0.52 (0.34, 0.81)	0.59 (0.33, 1.09)	0.69 (0.08, 5.66)	0.010

Mean ± SE; adjusted for age, body mass index, sex, energy, education, physical activity, marital status, smoking, drinking, hypertension, and diabetes mellitus; PP, Plant Protein; AP, Animal Protein.

## Data Availability

Detailed datasets about the surveys are available at https://knhanes.kdca.go.kr/knhanes/eng/main.do (accessed on 25 March 2026).

## Abbreviations

The following abbreviations are used in this manuscript:

AP	Animal protein
PP	Plant protein
RDI	Recommended dietary intake
KNHANES	Korea National Health and Nutrition Examination Survey

## References

[1] WHO Depression. https://www.who.int/news-room/fact-sheets/detail/depression.

[2] Ferriani L.O., Silva D.A., Molina M., Mill J.G., Brunoni A.R., da Fonseca M.J.M., Moreno A.B., Bensenor I.M., de Aguiar O.B., Barreto S.M. (2023). Depression is a risk factor for metabolic syndrome: Results from the ELSA-Brasil cohort study. J. Psychiatr. Res..

[3] Sevil-Perez A., Lopez-Anton R., Gracia-Garcia P., de la Camara C., Gascon-Catalan A., Santabarbara J. (2024). The Association Between Major Depression and Alzheimer’s Disease Risk: Evidence from a 12-Year Longitudinal Study. J. Clin. Med..

[4] Cai H., Xie X.M., Zhang Q., Cui X., Lin J.X., Sim K., Ungvari G.S., Zhang L., Xiang Y.T. (2021). Prevalence of Suicidality in Major Depressive Disorder: A Systematic Review and Meta-Analysis of Comparative Studies. Front. Psychiatry.

[5] Marx W., Lane M., Hockey M., Aslam H., Berk M., Walder K., Borsini A., Firth J., Pariante C.M., Berding K. (2021). Diet and depression: Exploring the biological mechanisms of action. Mol. Psychiatry.

[6] Suga H., Asakura K., Kobayashi S., Nojima M., Sasaki S., the Three-Generation Study of Women on Diets and Health Study Group (2018). Association between habitual tryptophan intake and depressive symptoms in young and middle-aged women. J. Affect. Disord..

[7] Li Y., Zhang C., Li S., Zhang D. (2020). Association between dietary protein intake and the risk of depressive symptoms in adults. Br. J. Nutr..

[8] Oh J., Yun K., Chae J.H., Kim T.S. (2020). Association Between Macronutrients Intake and Depression in the United States and South Korea. Front. Psychiatry.

[9] Forootani B., Sasanfar B., Salehi-Abargouei A., Mirzaei M. (2025). The association between plant and animal protein intake with depression, anxiety, and stress. Nutr. Neurosci..

[10] Dobersek U., Teel K., Altmeyer S., Adkins J., Wy G., Peak J. (2023). Meat and mental health: A meta-analysis of meat consumption, depression, and anxiety. Crit. Rev. Food Sci. Nutr..

[11] Song X., He K., Xu T., Tian Z., Zhang J., He Y., Fang J., Jiang K., Fan X., Tao Y. (2024). Association of macronutrient consumption quality, food source and timing with depression among US adults: A cross-sectional study. J. Affect. Disord..

[12] Sheikhi A., Siassi F., Djazayery A., Guilani B., Azadbakht L. (2023). Plant and animal protein intake and its association with depression, anxiety, and stress among Iranian women. BMC Public Health.

[13] Korea Disease Control and Prevention Agency The Korea National Health and Nutrition Examination Survey (KNHANES), 2016–2022. https://knhanes.kdca.go.kr/knhanes/eng/main.do.

[14] WHO Global physical activity questionnaire (GPAQ). https://www.who.int/teams/noncommunicable-diseases/surveillance/systems-tools/physical-activity-surveillance.

[15] Lee J., Lee C., Min J., Kang D.W., Kim J.Y., Yang H.I., Park J., Lee M.K., Lee M.Y., Park I. (2020). Development of the Korean Global Physical Activity Questionnaire: Reliability and validity study. Glob Health Promot..

[16] Piercy K.L., Troiano R.P., Ballard R.M., Carlson S.A., Fulton J.E., Galuska D.A., George S.M., Olson R.D. (2018). The Physical Activity Guidelines for Americans. JAMA.

[17] Kroenke K., Spitzer R.L. (2002). The PHQ-9: A new depression diagnostic and severity measure. Psychiatr. Ann..

[18] Kroenke K., Spitzer R.L., Williams J.B. (2001). The PHQ-9: Validity of a brief depression severity measure. J. Gen. Intern. Med..

[19] Kauffman K., Horvat Davey C., Dolata J., Figueroa M., Gunzler D., Huml A., Pencak J., Sajatovic M., Sehgal A.R. (2021). Changes in Self-Reported Depressive Symptoms Among Adults in the United States From 2005 to 2016. J. Am. Psychiatr. Nurses Assoc..

[20] Park S.J., Choi H.R., Choi J.H., Kim K.W., Hong J.P. (2010). Reliability and Validity of the Korean Version of the Patient Health Questionnaire-9 (PHQ-9). Anxiety Mood.

[21] Park S.-H., Kim S.-N., Lee S., Choe J.-S., Choi Y. (2018). Development of 9 th Revision Korean Food Composition Table and Its Major Changes. Korean J. Community Nutr..

[22] Joint WHO/FAO/UNU Expert Consultation (2007). Protein and Amino Acid Requirements in Human Nutrition.

[23] Ministry of Health and Welfare, The Korean Nutrition Society (2020). Dietary Reference Intakes for Koreans 2020 Sejong.

[24] Nucci D., Fatigoni C., Amerio A., Odone A., Gianfredi V. (2020). Red and Processed Meat Consumption and Risk of Depression: A Systematic Review and Meta-Analysis. Int. J. Environ. Res. Public Health.

[25] Zielinska M., Luszczki E., Deren K. (2023). Dietary Nutrient Deficiencies and Risk of Depression (Review Article 2018-2023). Nutrients.

[26] Migchelbrink M.M., Kremers S.H.M., den Braver N.R., Groeneveld L., Elders P.J.M., Blom M.T., Beulens J.W., Rutters F. (2024). The cross-sectional association between dietary total, animal, and plant-based protein intake and the prevalence and severity of depressive symptoms in Dutch adults with type 2 diabetes: The Hoorn Diabetes Care System cohort. Prev. Med..

[27] Reuter M., Zamoscik V., Plieger T., Bravo R., Ugartemendia L., Rodriguez A.B., Kirsch P. (2021). Tryptophan-rich diet is negatively associated with depression and positively linked to social cognition. Nutr. Res..

[28] Cowen P.J., Browning M. (2015). What has serotonin to do with depression?. World Psychiatry.

[29] Fernstrom J.D. (2013). Large neutral amino acids: Dietary effects on brain neurochemistry and function. Amino Acids.

[30] Bruncsics B., Hullam G., Bolgar B., Petschner P., Millinghoffer A., Gecse K., Eszlari N., Gonda X., Jones D.J., Burden S.T. (2023). Genetic risk of depression is different in subgroups of dietary ratio of tryptophan to large neutral amino acids. Sci. Rep..

[31] Thi Thu Nguyen T., Miyagi S., Tsujiguchi H., Kambayashi Y., Hara A., Nakamura H., Suzuki K., Yamada Y., Shimizu Y., Nakamura H. (2019). Association between Lower Intake of Minerals and Depressive Symptoms among Elderly Japanese Women but Not Men: Findings from Shika Study. Nutrients.

[32] Hajhashemy Z., Shirani F., Askari G. (2025). Dietary Magnesium Intake in Relation to Depression in Adults: A GRADE-Assessed Systematic Review and Dose-Response Meta-analysis of Epidemiologic Studies. Nutr. Rev..

[33] Anjom-Shoae J., Sadeghi O., Hassanzadeh Keshteli A., Afshar H., Esmaillzadeh A., Adibi P. (2018). The association between dietary intake of magnesium and psychiatric disorders among Iranian adults: A cross-sectional study. Br. J. Nutr..

